# Personalized B-cell tailored dosing of ocrelizumab in patients with multiple sclerosis during the COVID-19 pandemic

**DOI:** 10.1177/13524585211028833

**Published:** 2021-07-09

**Authors:** Zoë YGJ van Lierop, Alyssa A Toorop, Wouter JC van Ballegoij, Tom BG Olde Dubbelink, Eva MM Strijbis, Brigit A de Jong, Bob W van Oosten, Bastiaan Moraal, Charlotte E Teunissen, Bernard MJ Uitdehaag, Joep Killestein, Zoé LE van Kempen

**Affiliations:** Department of Neurology, MS Center Amsterdam, Amsterdam Neuroscience, Amsterdam UMC, Vrije Universiteit Amsterdam, Amsterdam, The Netherlands; Department of Neurology, MS Center Amsterdam, Amsterdam Neuroscience, Amsterdam UMC, Vrije Universiteit Amsterdam, Amsterdam, The Netherlands; Department of Neurology, MS Center Amsterdam, Amsterdam Neuroscience, Amsterdam UMC, Vrije Universiteit Amsterdam, Amsterdam, The Netherlands/Department of Neurology, OLVG Hospital, Amsterdam, The Netherlands; Department of Neurology, MS Center Amsterdam, Amsterdam Neuroscience, Amsterdam UMC, Vrije Universiteit Amsterdam, Amsterdam, The Netherlands/Department of Neurology, Canisius Wilhelmina Hospital, Nijmegen, The Netherlands; Department of Neurology, MS Center Amsterdam, Amsterdam Neuroscience, Amsterdam UMC, Vrije Universiteit Amsterdam, Amsterdam, The Netherlands; Department of Neurology, MS Center Amsterdam, Amsterdam Neuroscience, Amsterdam UMC, Vrije Universiteit Amsterdam, Amsterdam, The Netherlands; Department of Neurology, MS Center Amsterdam, Amsterdam Neuroscience, Amsterdam UMC, Vrije Universiteit Amsterdam, Amsterdam, The Netherlands; Department of Radiology & Nuclear Medicine, MS Center Amsterdam, Amsterdam Neuroscience, Amsterdam UMC, Vrije Universiteit Amsterdam, Amsterdam, the Netherlands; Department of Clinical Chemistry, Amsterdam Neuroscience, Amsterdam UMC, Vrije Universiteit Amsterdam, Amsterdam, The Netherlands; Department of Neurology, MS Center Amsterdam, Amsterdam Neuroscience, Amsterdam UMC, Vrije Universiteit Amsterdam, Amsterdam, The Netherlands; Department of Neurology, MS Center Amsterdam, Amsterdam Neuroscience, Amsterdam UMC, Vrije Universiteit Amsterdam, Amsterdam, The Netherlands; Department of Neurology, MS Center Amsterdam, Amsterdam Neuroscience, Amsterdam UMC, Vrije Universiteit Amsterdam, Amsterdam, The Netherlands

**Keywords:** Multiple sclerosis, ocrelizumab, personalized dosing, COVID-19, neurofilament light

## Abstract

In this observational study, 159 patients with multiple sclerosis received personalized dosing of ocrelizumab incentivized by the COVID-19 pandemic. Re-dosing was scheduled when CD19 B-cell count was ⩾10 cells/µL (starting 24 weeks after the previous dose, repeated 4-weekly). Median interval until re-dosing or last B-cell count was 34 [30–38] weeks. No clinical relapses were reported and a minority of patients showed Expanded Disability Status Scale (EDSS) progression. Monthly serum neurofilament light levels remained stable during extended intervals. Two (1.9%) of 107 patients with a follow-up magnetic resonance imaging (MRI) scan showed radiological disease activity. Personalized dosing of ocrelizumab could significantly extend intervals with low short-term disease activity incidence, encouraging future research on long-term safety and efficacy.

## Introduction

Ocrelizumab is an effective anti-CD20 therapy for patients with multiple sclerosis (MS).^1^ Since B-cell depletion is associated with an increased risk of infections, COVID-19 might be more severe in patients using anti-CD20 therapies.^2^

International MS experts advised neurologists to consider interval extension between ocrelizumab doses based on CD19 B-cell count.^3^ The Dutch MS Task Force recommended re-dosing of ocrelizumab after CD19 B cells re-emerged to ⩾10 cells/µL. The objectives of this study were to assess efficacy of personalized dosing of ocrelizumab and present the personalized dosing protocol as implemented at the MS Center Amsterdam during the COVID-19 pandemic.

## Methods

The protocol for personalized dosing of ocrelizumab was implemented as standard care from 15 March 2020 for all patients. All patients with MS receiving personalized dosing of ocrelizumab until 1 November 2020 who provided informed consent for the use of data were included. Data were prospectively collected. The routine blood draw scheduled 24 weeks after the previous 600 mg ocrelizumab dose was defined as the start of the personalized dosing protocol and included CD19 B-cell count measured by flow cytometry (Gallios Flow Cytometer, Beckman Coulter). Beads were used as calibrator (BD Trucount, BD Biosciences). On average 5.000 to 10.000 lymphocytes were counted. After a 300 mg ocrelizumab dose, the blood draw was scheduled after 12 weeks. The next ocrelizumab dose was withheld when CD19 B-cell count was below 10 cells/µL. Follow-up CD19 B-cell counts were repeated every 4 weeks. Re-dosing was scheduled within 2 weeks after CD19 B cells re-emerged to ⩾10 cells/µL. This cut-off was based on expert opinion on adequate B-cell depletion. Concurrent serum neurofilament light (sNfL) levels were measured every 4 weeks during extended intervals (Simoa Advantage Kit, Quanterix). Relapses (defined as new neurological symptoms evaluated by a neurologist with a duration of more than 24 hours and not caused by other factors than MS) were assessed at the start of the personalized dosing protocol and 4-weekly onwards until re-dosing by telephone and, in case of new neurological symptoms, by physical examination. After re-dosing, relapses were assessed at the annual visit with a treating neurologist and recorded until database closure on 1 November 2020. Patients received annual brain magnetic resonance imaging (MRI) scans according to international guidelines.^4^ We applied linear regression (correcting for age and body mass index (BMI)) and mixed models (correcting for within-subjects correlations) to assess the effect of extended intervals on log-transformed sNfL levels (SPSS version 26). Approval of the institutional ethics committee was obtained (Amsterdam UMC Ethics Committee nos 2020.269 and 2016.554), as well as written informed consent from all participants.

## Results

During the observation period of this study, a total of 179 patients with MS were treated with ocrelizumab at our center. Seven patients who started ocrelizumab during this period were excluded from personalized dosing because of recent disease activity under previous disease-modifying therapy (DMT) and three patients were lost to follow-up. The remaining 169 patients received personalized dosing of ocrelizumab between 15 March 2020 and 1 November 2020, of whom 165 (98%) provided written informed consent for the use of data (Table 1) and were therefore included in this study. Patients were observed for a median of 45 [38–51] weeks from the previous ocrelizumab dose until 1 November 2020.

**Table 1.** Baseline characteristics and follow-up of the personalized dosing ocrelizumab cohort.

Baseline characteristics	Total (*n* = 165)
Age, years	42.8 ± 10.9
Gender, female	103 (62)
Body weight, kg	74.5 ± 12.6
Body mass index, kg/m^2^	24.3 ± 3.8
Type of MS	
RRMS	127 (77)
PPMS	31 (19)
SPMS	8 (4)
Previous DMT before start ocrelizumab	
None	39 (23.6)
Dimethylfumaric acid	39 (23.6)
Natalizumab	31 (18.8)
Fingolimod	14 (8.5)
Interferons	13 (7.9)
Glatiramer acetate	10 (6.1)
Teriflunomide	10 (6.1)
Daclizumab	5 (3)
Rituximab	3 (1.8)
Cladribine	1 (0.6)
Reasons for initiation/switch to ocrelizumab	
Disease progression including PPMS	32 (19.4)
Disease activity under previous DMT	86 (52.1)
High JC virus titer under natalizumab treatment	26 (15.8)
Neutralizing antibodies previous DMT	2 (1.2)
Side effects previous DMT	11 (6.7)
Discontinuation of rituximab or daclizumab	8 (4.8)
Time since diagnosis, years	9.9 [4.9–14.5]
EDSS score^a^	4.0 [2.5–5.0]
Duration of ocrelizumab treatment, months	16.7 [11.5–19.3]
Number of ocrelizumab doses	4 [3–4]
Radiological activity on MRI scan prior to visit for personalized dosing^b^	53 (32)
Median CD19 B-cell count, cells/µL	2 [1–7]
Median sNfL level, pg/mL	7.8 (6.1–10.8)

Follow-up	
Clinical relapses^c^	0 (0)
Radiological activity on MRI scan during follow-up^d^ (*n* = 107)	2 (1.9)
EDSS score^e^	4.0 [3.0–6.0]
Median CD19 B-cell count, cells/µL	15 [4–27]
Median sNfL level, pg/mL	8.2 (5.7–11.0)

RRMS: relapsing remitting multiple sclerosis; PPMS: primary progressive multiple sclerosis; SPMS: secondary progressive multiple sclerosis; sNfL: serum neurofilament light.

The start of the personalized dosing protocol (first blood draw after the previous dose) was defined as the baseline time point. Values are presented as numbers and percentage (%), mean values ± SD, or medians [IQR].

a The Expanded Disability Status Scale (EDSS) was assessed on a yearly basis. Before the COVID-19 pandemic, EDSS was performed after administration of the previous ocrelizumab dose. During the COVID-19 pandemic in 2020, most EDSS scores were assessed by telephone.

b Brain MRI scans were performed within 6 months after the start of ocrelizumab in 52 out of 53 (98%) of patients with radiological disease activity (T2 lesions and/or gadolinium-enhancing lesions) before start of the personalized dosing protocol (re-baseline scan).

c Clinical relapses were defined as new neurological symptoms evaluated by a neurologist with a duration of more than 24 hours and not caused by other factors than MS.

d Brain MRI scans were performed in 107 of 165 patients (65%) during follow-up. Among these 107 patients, 68 patients (64%) had a brain MRI scan during the personalized dosing protocol before re-dosing of ocrelizumab with a median follow-up of 25 [20–29] weeks from the previous ocrelizumab infusion, and 39 patients (36%) had a brain MRI scan after re-dosing of ocrelizumab with a median follow-up of 46 [35–52] weeks from the previous ocrelizumab infusion. Two patients (1.9%) had T2 lesions (*n* = 1) or gadolinium-enhancing lesions (*n* = 1) without evidence of radiological disease activity on the previous MRI scan. Ten (9.3%) other patients had evidence of radiological disease activity (new/enlarged T2 lesions) on the first brain MRI scan after the start of ocrelizumab and were evaluated as re-baseline scans.

e Follow-up EDSS scores were assessed yearly and available in 96 patients, of whom 23 patients (24%) showed EDSS progression (defined as a 1.5, 1, or 0.5 point increase in case of a reference EDSS of 0, 1–5, or ⩾5.5, respectively) compared to the most recent EDSS prior to the start of personalized dosing of ocrelizumab. Four patients were diagnosed with PPMS, two with SPMS, and eighteen with RRMS. The EDSS progression was due to a pseudo-exacerbation in one RRMS patient. The majority of patients in whom EDSS progression was observed during follow-up already showed some progression prior to personalized dosing.

**Figure 1.**
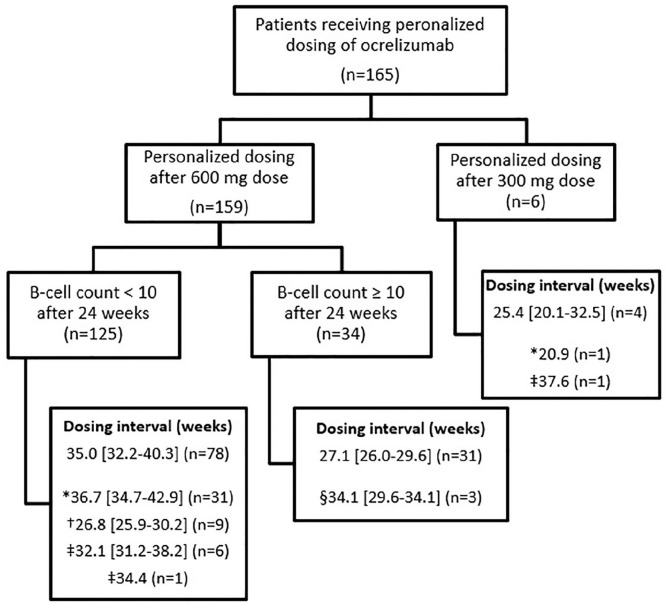
Flowchart of patients receiving personalized dosing of ocrelizumab. The number of patients (*n*) is indicated between brackets. Intervals are presented in median number of weeks with interquartile range [IQR] and were calculated between the previous ocrelizumab dose and re-dosing. Separate dosing intervals are presented for: *Patients whose CD19 B-cell counts remained <10 cells/µL during follow-up and the interval was calculated between previous dose and last follow-up on 1 November 2020 (*n* = 31 in personalized dosing after 600 mg dose and *n* = 1 in personalized dosing after first 300 mg dose). †Patients who requested the next dose of ocrelizumab regardless of CD19 B-cell count <10 cells/µL due to fear of recurrent disease activity or practical reasons (*n* = 9). ‡Patients who initially agreed to personalized dosing and later requested continuation of ocrelizumab regardless of CD19 cell count due to fear of recurrent disease activity or practical reasons (*n* = 6 in personalized dosing after 600 mg dose and *n* = 1 after 300 mg dose, and *n* = 1 who switched to another DMT due to personal preferences). §Patients who requested extended dosing of ocrelizumab in spite of CD19 B-cell count ⩾10 cells/µL, due to fear of immunosuppressive effects or hospital visits during the COVID-19 pandemic (*n* = 3).

Median CD19 B-cell count was 2 [1–7] cells/µL at the start of the personalized dosing protocol and 15 [4–27] cells/µL before re-dosing. In the cohort of 159 patients who received personalized dosing after a 600 mg dose, median interval duration between two consecutive doses or end of follow-up was 34 [30–38] weeks (Figure 1). Median sNfL levels at the start of personalized dosing were similar to sNfL levels prior to re-dosing (Table 1). We found no significant intra-individual changes in sNfL during extended intervals. Interval duration corrected for age and BMI did not significantly predict sNfL level prior to re-dosing.

No clinical relapses were observed and no corticosteroids were administered in all patients during the observation period. Brain MRI scans were performed in 107 of 165 patients (65%). Among these 107 patients, two patients (1.9%) had T2 lesions (*n* = 1) or gadolinium-enhancing lesions (*n* = 1) without evidence of radiological disease activity on the previous MRI scan. The first patient with relapsing remitting MS had a B-cell count of 1 CD19 B cell/µL at the start of personalized dosing (102 weeks after initiation of ocrelizumab therapy) and 15 cells/µL prior to re-dosing after a dosing interval of 34 weeks (sNfL level was 10.6 pg/mL; previous levels were 18.3 and 12.4 pg/mL; 95% prediction interval in healthy controls of similar age is 4–46 pg/mL). The MRI scan performed without gadolinium 15 weeks after re-dosing showed two new cerebral T2 lesions. The MRI scan performed a year prior to the follow-up scan showed no disease activity. The second patient diagnosed with primary progressive MS had a B-cell count of 1 CD19 B cells/µL at the start of personalized dosing (54 weeks after initiation of therapy with ocrelizumab). The MRI scan after interval extension to 35 weeks showed one small new gadolinium-enhancing lesion located periventricular at the posterior horn of the left lateral ventricle. By then, CD19 B-cell count was 3 cells/µL and sNfL level was 8.9 pg/mL. The MRI scan performed 1.5 years prior to the follow-up scan showed no disease activity. The patient experienced worsening of symptoms prior to personalized dosing in the past year with increased paresis of the right leg, but did not experience clinical exacerbations. Interval extension was continued until CD19 B-cell count re-emerged to 11 cells/µL after 45 weeks (sNfL level was 10.3 pg/mL; 95% prediction interval in healthy controls of similar age is 3–29 pg/mL).

## Discussion

In this observational cohort of B-cell tailored personalized dosing of ocrelizumab, dosing intervals were extended in the majority of patients. Two patients (1.9%) had evidence of new radiological disease activity, a relatively low proportion, roughly comparable to disease activity previously observed in phase III trials.^1^

Although personalized therapy with monoclonal antibodies is evolving fast,^5^ the literature on B-cell tailored personalized treatment in MS is limited. In phase II trials of ocrelizumab, repopulation of CD19 B cells to the lower limit of normal occurred 72 weeks after the previous 600 mg dose, suggesting a dosing interval of 24 weeks is relatively short.^6^ A recent study suggests the dosing interval of ocrelizumab could be extended while maintaining efficacy, with fewer adverse events and possibly a more effective vaccination response due to recovery of immature B cells.^7^ Another recent retrospective study in a smaller cohort of 33 patients reported interval extension of ocrelizumab to 33 ± 2.7 weeks during the COVID-19 pandemic, which is comparable to our results.^8^

This study provides an overview of personalized dosing of ocrelizumab in a large, closely monitored cohort. Recurrence of disease activity was monitored by monthly sNfL in all patients. In the single patient showing a gadolinium-enhancing lesion in this study, sNfL remained low, which is unexpected given the claim that normal sNfL levels strongly argue against MS disease activity.^9^ An explanation could be the small size of this lesion. Furthermore, the optimal cut-off for B cells is still unknown and should be evaluated in future studies. Limitations of this study include the short follow-up and observational design, and as such not all patients received a follow-up brain MRI scan or received the brain MRI scan during standard interval dosing. Also, one should carefully interpret the Expanded Disability Status Scale (EDSS) findings, as follow-up scores were not available in all patients and changes in EDSS were not confirmed at 3 or 6 months. In addition, clinical or radiological disease activity could occur after the follow-up period of this study.

In conclusion, personalized dosing of ocrelizumab based on serum CD19 B-cell count incentivized by the COVID-19 pandemic led to an interval extension in the majority of patients with a low short-term incidence of disease activity, encouraging future studies to confirm safety and efficacy of this protocol with a longer follow-up period beyond the COVID-19 pandemic.
